# Pooled Cohort Equations and the competing risk of cardiovascular disease versus cancer: Multi-Ethnic study of atherosclerosis

**DOI:** 10.1016/j.ajpc.2021.100212

**Published:** 2021-06-14

**Authors:** Seamus P. Whelton, Catherine Handy Marshall, Miguel Cainzos-Achirica, Omar Dzaye, Roger S. Blumenthal, Khurram Nasir, Robyn L. McClelland, Michael J. Blaha

**Affiliations:** a Johns Hopkins Ciccarone Center for Prevention of Cardiovascular Disease, 600 North Wolfe Street, Blalock 524A, Baltimore, MD 21287, United States; b Sidney Kimmel Comprehensive Cancer Center, Baltimore, MD, United States; c Division of Cardiovascular Prevention and Wellness, Department of Cardiology, Houston Methodist DeBakey Heart and Vascular Center, Houston, TX, United States; d Department of Biostatistics, University of Washington, United States

**Keywords:** Cardiovascular disease, Cancer, Risk prediction, Competing risks, Screening

## Abstract

**Background:**

many of the modifiable variables in the Pooled Cohort Equations (PCE) are shared risk factors for cardiovascular disease (CVD) and cancer, which are the two leading causes of death in the United States. We sought to determine the utility of the PCE risk for the synergistic risk prediction of CVD and cancer.

**Methods:**

we identified 5,773 participants (61.5 years and 53% women) without baseline CVD or cancer from the Multi-Ethnic study of atherosclerosis. The primary outcome was time to first event of either incident CVD or incident cancer. We calculated competing risk and cause-specific hazard models to examine the association of the PCE groups (<7.5%, 7.5–<20%, ≥20%) with the competing risk of CVD and cancer.

**Results:**

the rate of incident CVD and cancer was higher with higher PCE risk, but the absolute event rate was low for both CVD and cancer when the PCE risk was <7.5%. Participants with a PCE <7.5% had a higher rate of cancer (4.8) compared to CVD (3.3) per 1000 person-years, while the rate of CVD (11.5) was higher than cancer (8.6) for PCE between 7.5 and <20%. The ratio of CVD to cancer increased in a logarithmic manner and at a PCE risk of approximately 7.2% the risk for CVD and cancer was equal. In adjusted competing risk modeling, a PCE risk of ≥20% compared to <7.5% was associated with a greater risk of both CVD [7.18 (95% CI 5.77–8.94)] and cancer [3.59 (95% CI 2.91–4.43)].

**Conclusions:**

these findings highlight the importance of age and modifiable risk factors for CVD and cancer prevention. In addition, it suggests that the PCE can provide important information for both CVD and cancer risk stratification, which may guide a synergistic approach to screening and preventive therapies for the two leading causes of death in the United States.

## Graphical abstract

**Figure:**
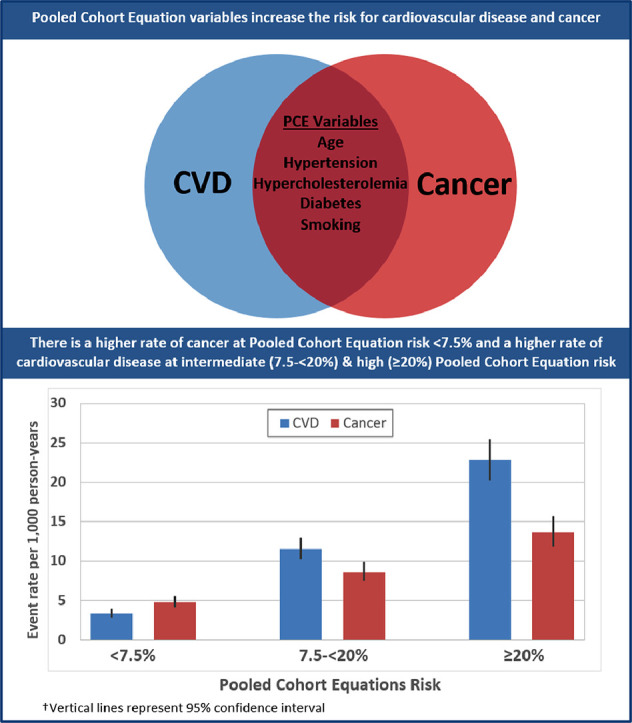

## 1 Introduction

The overall rate of cardiovascular disease (CVD) mortality has declined dramatically over the last four decades, while the rate of non-CVD mortality such as cancer has remained relatively constant [1]. Accordingly, the rate of CVD and cancer mortality in the United States are currently very similar [2]. In fact, cancer is already the leading cause of death in 22 of the United States along with 9 European countries and it has been predicted that cancer may become the overall leading cause of death [1,[3], [4], [5]]. With this decline in CVD, there is an increase in competing risks and this has important implications for individual risk prediction [6,7].

The 2018 American Heart Association (AHA) / American College of Cardiology (ACC) Cholesterol Treatment Guidelines and the 2019 ACC/AHA Primary Prevention of CVD Guidelines are based upon the Pooled Cohort Equations (PCE) individual 10-year atherosclerotic cardiovascular disease (ASCVD) risk estimate. Many of the modifiable variables in the PCE such as hypertension, cholesterol, smoking, and diabetes are shared risk factors for CVD and cancer [[8], [9], [10], [11], [12], [13], [14], [15], [16]]. Indeed, Pursnani et al have demonstrated that individuals in the ACC/AHA statin eligibility groups have an increased risk of both incident CVD and cancer mortality [17]. However, CVD and cancer are typically treated as separate disease processes and a better understanding the shared epidemiology to facilitate risk prediction and screening has been identified as a key area of future research [18].

Despite the many shared modifiable risk factors, the risk of incident CVD and cancer as a function of the PCE has not previously been described and there is currently no information on: (1) the proportion of individuals expected to develop an incident cancer event over the next 10 years within low, intermediate, and high risk PCE groups, (2) how the incident cancer rate changes as a function of the PCE-derived risk, and (3) the PCE risk at which an individual is more likely to first experience incident CVD versus incident cancer. A better understanding of how the risk for incident CVD versus incident cancer changes as a function of the PCE risk may provide clinically meaningful insight into the utility of the PCE for the synergistic approach to screening and prevention strategies for CVD and cancer.

## 2 Methods

### 2.1 Study population

The Multi-Ethnic study of atherosclerosis (MESA) is a community-based cohort comprised of adult participants age 45–84 years old free of CVD at baseline that has previously been described in detail [19]. Participants were excluded from this analysis if they had known cancer at baseline (based on self-report) (*n* = 543), or other diseases suggestive of possible underlying undiagnosed cancer including emphysema (*n* = 90), liver disease (*n* = 210), prior blood clots (*n* = 119). We also excluded participants with an unknown or un-adjudicated cause of death (*n* = 31) or if they were missing information to calculate their PCE risk (*n* = 48), which resulted in a total of 5,773 participants. The study protocol was approved by the institutional review boards at each of the six MESA field centers.

### 2.2 Outcome ascertainment

The primary outcomes for this analysis were the time to first event of either (1) incident CVD (incident or fatal coronary heart disease (myocardial infarction, resuscitated cardiac arrest, fatal coronary heart disease, and coronary revascularization only if the participant also had prior or concurrent adjudicated angina), incident or fatal stroke, and other incident and or fatal ASCVD) and (2) incident cancer. Participants were removed from the dataset after the diagnosis of either incident CVD or incident cancer. Therefore, the results should be interpreted as the risk for developing CVD (or cancer) before a diagnosis of cancer (or CVD). Participants or their family were contacted by telephone on every 9 to 12 months and asked about any new hospitalizations, new outpatient diagnoses, procedures, or death that had occurred since the last telephone interview. All reported CVD events were adjudicated by two physicians according to pre-defined criteria using hospital records and medical records [20]. The diagnosis of incident cancer was made based on review of all available inpatient hospital records and the presence of an associated ICD-9 code between 140 and 209 [21]. If a diagnosis of incident CVD and incident cancer were reported during the same telephone interview for 16 participants. In these instances we coded the incident CVD event as the primary (first) outcome of interest.

### 2.3 Risk factors

The participants’ PCE risk was calculated using the 2013 ACC/AHA PCE and the scores were categorized as low/borderline <7.5%, intermediate 7.5-<20%, and high ≥20% based on the 2018 ACC/AHA Cholesterol Guidelines [8,22]. Diabetes was defined as the use of a blood glucose lowering medication or a fasting blood glucose ≥126 mg/dL. Smoking was defined as the current use of cigarettes. Socioeconomic status was estimated based on the highest obtained education and annual household income.

### 2.4 Statistical analysis

We calculated incident CVD and cancer event rates per 1000 person-years of follow-up stratified by PCE-defined risk categories. We then calculated the ratio of incident CVD events per 1000 person-years to incident cancer events per 1000 person-years as a function of PCE risk deciles and fitted these results using a logarithmic function. Cox proportional hazard models overestimate the risk relationship when there are competing risks [7,[23], [24], [25]]. Therefore, we calculated the cumulative incident function (CIF), which accounts for competing risk, unlike Kaplan-Meier survival analysis, for incident CVD and cancer stratified by PCE risk group with the results displayed as a survival curve (1-CIF) [26]. We also calculated Cox Proportional cause-specific hazards models, which also take into account competing risks [27]. In order to use the most appropriate the follow-up time for our outcomes of interest: (1) we used the Fine and Gray approach for calculating follow-up time for the incident event rates and CIF, because it best for estimating the number or proportion of events that have occurred, and (2) a Cox Proportional Hazards cause-specific approach for calculating follow-up time for the relative association between the PCE and incident CVD versus cancer.

We first report unadjusted models, because the PCE risk already incorporates age, sex, and traditional CVD risk factors. We also report the results of an adjusted model that includes other CVD risk modifiers not incorporated into the PCE risk including lipid-lowering medication use, body mass index, and socioeconomic status. In addition, we calculated cause-specific hazards models stratified by age <65 and ≥65 years, because age is one of the predominant contributors to an individual's estimated PCE risk, especially among individuals ≥65 years old [28]. We also performed sex-stratified analyses and a sensitivity analysis excluding participants who were prescribed lipid lowering medications (*n* = 918). In addition, we performed race/ethnicity specific analyses. We evaluated discrimination using Harrell's C-statistic and the calibration slope (linear regression of observed versus predicted events) was used to evaluate calibration.

## 5 Results

The mean age was 61.5 years, 53% of participants were women, and 36% were Caucasian (Table 1). Over the mean follow-up time of 11.3 (SD 3.7) years there were 715 incident CVD events and 613 incident diagnoses of cancer. 113 participants developed both CVD and cancer and of those 68 (60%) developed CVD before cancer. The prevalence of traditional CVD risk factors except for LDL-C and total cholesterol increased with an increasing PCE risk (Table 1). The mean PCE risk was 12.8% and 78% of participants had a PCE risk of <20%. The most common types of incident cancer were genitourinary, digestive organs, respiratory, and breast (Supplemental Fig. 1).

**Table 1:** Participant characteristics.

	Total cohort(*n* = 5,773)	PCE <7.5%(*n* = 2,601)	PCE 7.5-<20% (1,887)	PCE ≥20%(*n* = 1,285)	*p* for trend
Age (years)	61.5 (10.1)	53.9 (6.3)	64.3 (7.4)	72.8 (6.6)	<0.001
Male	47.3	33.9	56.0	61.5	<0.001
Race/Ethnicity					<0.001
Caucasian	36.0	40.5	32.6	31.9	<0.001
Black	28.6	22.2	36.1	30.7	<0.001
Hispanic	23.2	24.3	20.3	25.4	0.01
Chinese	12.1	13.1	10.9	12.1	0.09
Systolic Blood Pressure (mmHg)	126.3 (21.3)	115.4 (15.6)	129.3 (18.8)	143.9 (21.4)	<0.001
Diastolic Blood Pressure (mmHg)	72.1 (10.2)	69.9 (9.5)	73.5 (10.2)	74.7 (10.6)	<0.001
Anti-hypertensive therapy	36.2	17.9	43.0	63.3	<0.001
LDL-C (mg/dL)	118.2 (31.6)	117.6 (30.5)	119.2 (32.5)	117.8 (32.6)	0.90
HDL-C (mg/dL)	50.8 (14.8)	52.9 (15.0)	49.7 (14.5)	48.3 (14.2)	<0.001
Total Cholesterol (mg/dL)	195.1 (34.8)	195.0 (34.4)	195.7 (35.9)	194.1 (38.3)	0.19
Lipid lowering therapy	15.9	10.1	19.0	35.6	<0.001
Diabetes	12.6	3.5	12.3	26.8	<0.001
Current Smoking	12.8	9.9	15.6	14.7	<0.001
Family History heart disease	41.9	39.9	43.4	44.0	<0.001
Body Mass Index (kg/m^2^)	28.3 (5.4)	28.1 (5.7)	28.7 (5.3)	28.2 (5.0)	0.007
ASCVD Score (%)	12.9 (12.8)	3.4 (2.0)	12.8 (2.2)	32.4 (11.9)	<0.001

Value reported as mean (SD) or percent unless otherwise noted.

*ASCVD: Atherosclerotic cardiovascular disease.

**Fig. 1:**
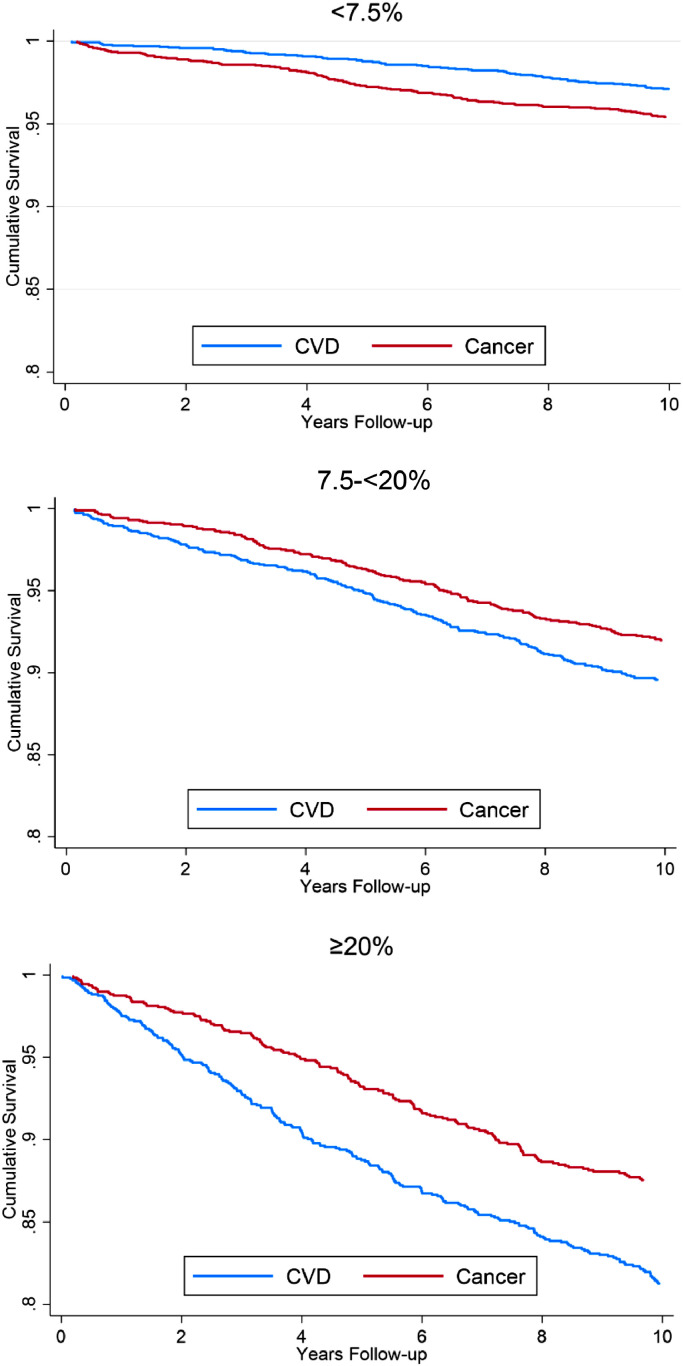
Cumulative survival free from incident cardiovascular disease or cancer stratified by 10-year atherosclerotic cardiovascular disease risk group.

**Fig. 2:**
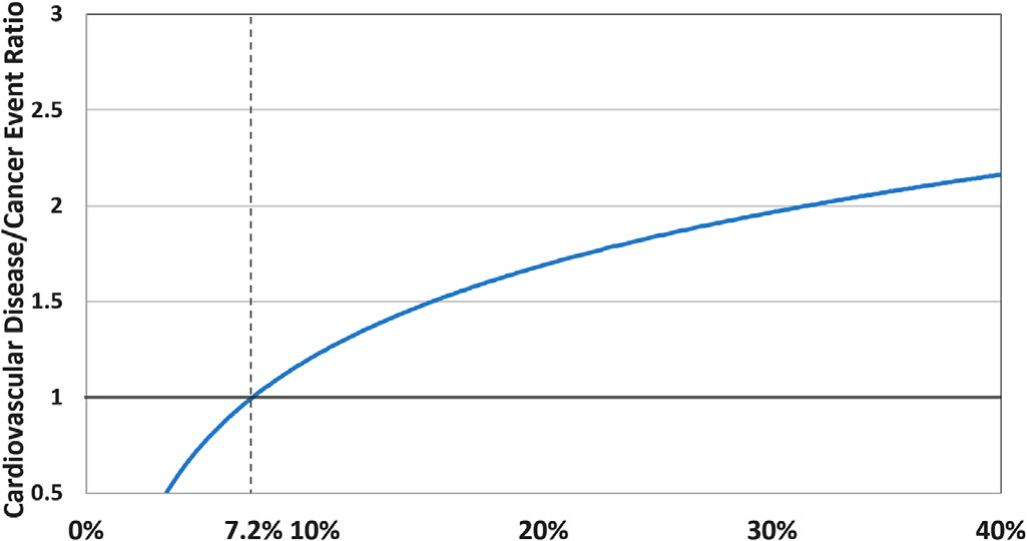
Ratio of incident cardiovascular disease event rate to incident cancer event rate per 1,000 person-years follow-up as a function of atherosclerotic cardiovascular disease risk.

Among individuals with a PCE risk <7.5% there was a higher proportion who developed incident cancer (6.2%) versus incident CVD (4.3%). The proportion of individuals with incident CVD versus incident cancer was higher when the PCE risk was between 7.5-<20% (CVD 13.6%, cancer 10.4%) and when it was ≥20% (CVD 23.3%, cancer 14.8%). Both the CVD and cancer event rate also increased with increasing PCE risk and the absolute event rate was low for both CVD and cancer when the PCE risk was <7.5% (Central illustration). For individuals with a PCE risk score <7.5% the cumulative survival free from CVD was greater than that of cancer, but for individuals with a PCE risk of ≥7.5% the cumulative survival free from cancer was greater than CVD (Fig. 1). Among individuals with PCE risk <7.5% there was a higher proportion who developed incident cancer (6.9%) versus incident CVD (4.7%), but when the PCE risk was between 7.5-<20% there was a higher proportion of individuals with incident CVD (17.2%) versus incident cancer (13.6%). Individuals with a <7.5% PCE risk had a higher rate of incident cancer (4.8) versus incident CVD (3.3) per 1,000 person-years follow-up and the incidence rate of CVD overtook cancer in the PCE risk ≥7.5% group (Supplemental Table 1). The ratio of incident CVD to cancer events increased logarithmically and the ratio was higher for CVD after a PCE risk of approximately 7.2% (Fig. 2).

Compared to individuals with a PCE risk <7.5%, there was a significantly increased risk of incident CVD cause-specific hazard (3.56, 95% CI 2.86–4.43) and of incident cancer (2.10, 95% CI 1.71–2.57) for individuals with a PCE risk of 7.5-<20%. Individuals with a PCE risk ≥20% had a greater than sevenfold increased risk for CVD and greater than threefold increased risk for cancer (Table 2). For individuals <65 years of age with a PCE risk ≥20% there was a significantly increased risk of incident CVD cause-specific hazard 6.97 (95% CI 4.69–10.37) and incident cancer with a cause-specific hazard of 2.17 (95% CI 1.29–3.67) compared to those with a PCE risk <7.5% (Supplemental Table 2). For individuals ≥65 years of age with a PCE risk ≥20% there was a significantly and similarly increased risk of both incident CVD cause-specific hazard 3.35 (95%2.08–5.40) and incident cancer cause-specific hazard 2.94 (95% CI1.74–4.98) compared to those with a PCE risk <7.5%. The results were similar for women compared to men, when participants prescribed lipid lowering medications were excluded from the analysis, and across the four race/ethnicities included in MESA. The PCE had a C- statistic of 0.73 CVD and 0.65 cancer. The calibration of the PCE for both CVD and cancer was good in the risk ranges normally encountered in clinical practice (Supplemental Fig. 2).

**Table 2:** Cause-specific hazard for the competing risk of CVD and cancer by ASCVD group.

Pooled Cohort Equation	<7.5%	7.5-<20%	≥20%
Cardiovascular			
Events	113	257	300
Unadjusted	Reference	3.72 (2.99-4.62)	7.60 (6.14-9.40)
Model 1	Reference	3.56 (2.86-4.43)	7.18 (5.77-8.94)
Cancer			
Events	161	197	186
Unadjusted	Reference	2.09 (1.71–2.56)	3.49 (2.85–4.28)
Model 1	Reference	2.10 (1.71–2.57)	3.59 (2.91–4.43)

Model 1 – Lipid-lowering medication use, body mass index, income, education.

## 6 Discussion

These results demonstrate a higher risk for incident cancer when the PCE risk was <7.2% and above this score the risk of incident CVD overtook the risk for cancer. However, the cumulative incidence of both incident CVD and incident cancer was higher with higher PCE risk. Most importantly, these findings demonstrate the clinical utility of the PCE as a synergistic tool for not only CVD, but also cancer risk stratification.

CVD and cancer are the two leading causes of death in the United States and Europe, but despite the many shared modifiable risk factors current approaches to screening and prevention are separate and generally performed in isolation by primary care providers, cardiologists, and oncologists [2,18,29]. While a synergistic approach to CVD and cancer prevention has been proposed, to date there is no single harmonized risk prediction algorithm [16]. Our results build upon the knowledge that statin eligible individuals have an increased risk of cancer by describing (1) how the rate of CVD and cancer change as a function of PCE risk and (2) the approximate PCE risk at which incident CVD becomes more likely than cancer (e.g. 7.2%), the latter of which may be helpful to providers and patients who may wish to know whether they are more likely to develop incident CVD versus incident cancer along with epidemiologists interested in modeling the risk of CVD versus cancer [17]. These findings suggest that a more focused approach to CVD and cancer prevention strategies could be considered when the PCE risk is very low or very high. However, while incident CVD overtakes cancer at a PCE risk of approximately 7.2%, the cumulative incidence of cancer continues to increase beyond a PCE risk of 7.2%. For instance, among individuals with a PCE risk ≥20% the 10-year cumulative incidence of cancer was 12% versus 18% for CVD. Therefore, even though individuals with a PCE risk ≥20% are at a higher risk for incident CVD than cancer, age appropriate cancer screening should still be strongly recommended based on their concomitantly elevated absolute risk for cancer.

Calculation of the PCE is already guideline recommended for CVD risk stratification [8]. It is routinely used in clinical practice by both cardiologists and primary care providers and providing a PCE-based cancer estimate requires no additional burden to the clinician or patient. While cancer screening strategies largely use an age-based rather than risk-based approach to screening, the recommended ages for the screening of common cancers corresponds to the age group in which PCE risk calculation is recommended for CVD risk stratification [[30], [31], [32], [33]]. Providing a PCE-based cancer risk estimate may be especially pragmatic among individuals without recent age-appropriate cancer screening (approximately 30% of US adults for breast cancer and 40% for colorectal cancer) as the knowledge of an elevated PCE-based cancer risk may be especially informative in their cancer screening decision-making process [34]. Accordingly, interpretation of the PCE risk for only CVD risk stratification represents an incomplete utilization of the data already at hand.

Aspirin is currently the only medication with guideline-based recommendation for dual CVD and cancer prevention. This shared indication from the 2016 United States Preventive Services Task Force Preventive Services is specifically for the prevention of CVD and colorectal cancer among persons 50 to 59 years old with low bleeding risk and PCE risk ≥10% [35]. However, the 2019 ACC/AHA Guideline on the Primary Prevention of CVD provides only a IIb recommendation for aspirin and primary prevention of ASCVD among persons with higher PCE risk [36]. Aspirin has also been shown to reduce the incidence and/or mortality associated with breast, lung, and other gastrointestinal cancers [[37], [38], [39], [40]]. There is also growing evidence demonstrating that several other CVD medications can reduce the risk of cancer. Canakinumab reduced both recurrent CVD events along with total cancer mortality (particularly lung cancer) in the Canakinumab Anti-inflammatory Thrombosis Outcome Study (CANTOS) trial and is under investigation for the treatment for lung cancer [41,42]. Most relevant to calculation of the PCE, statin use is associated with a significant reduction in the incidence and/or mortality of hepatocellular, breast, prostate, kidney, colorectal, and lung cancers [[43], [44], [45], [46], [47], [48], [49], [50]].

Age is a very strong contributor to an individual's PCE risk, especially among older individuals as nearly all individuals ≥65 years of age have a PCE risk ≥7.5%, regardless of their presence or absence of other CVD risk factors [51]. In our subgroup analysis we found that the PCE had a much lower relative hazard for CVD in older compared to younger individuals. However, in older individuals with an intermediate PCE risk, the relative hazard for cancer was higher than CVD, the latter of which showed a statistically non-significant association, HR 1.58 (95% CI 0.97–2.59). Accordingly, our results suggest that among older patients at intermediate PCE risk, an especially strong emphasis should be placed upon age appropriate cancer screening in addition to the appropriate CVD preventive therapies. Coronary artery calcium (CAC) scoring, which is recommended by the 2018 ACC/AHA Cholesterol Treatment Guidelines when there is uncertainty among intermediate risk individuals can also be considered in this group as we have previously demonstrated that CAC can refine the risk of CVD versus cancer mortality [15,52]. Further research is necessary to better understand these observed differences in the competing risk of CVD and cancer by age and how they may impact primary prevention strategies.

Limitations of this analysis include that unlike CVD, incident cancer events were not adjudicated in MESA. While some participants likely had undetected or undiagnosed cancer, a diagnosis of cancer is not typically made in clinical practice without tissue pathology and it is unlikely that there were many, if any false positive diagnoses. However, participants diagnosed with cancer who did not have any subsequent hospitalizations would have been classified as without a cancer event in this study, although cancers not requiring hospitalization for treatment are less likely to be associated with significant adverse clinical outcomes. A general limitation of the PCE is that older individuals are much more likely to be classified as high-risk and although we explored the effect of age in a subgroup analysis future more detailed analyses examining the impact of age and sex are needed. Additionally, the categorical cutpoints of <7.5%, 7.5–19%, and ≥20% for the PCE are based on risk benefit data for statin use and not optimized for cancer screening. Additional research is needed to determine the optimal ASCVD risk cutpoints for consideration of CVD versus cancer in clinical medicine. Strengths include that MESA is a well-defined, multiethnic cohort of individuals in an age group for which the use of the PCE is guideline and cancer screening is recommended.

## 7 Conclusions

At low PCE risk there was a higher incidence of cancer versus CVD incident rate with CVD overtaking cancer at a PCE of approximately 7.2%. However, the absolute event rate was low for both CVD and cancer when the PCE risk was <7.5%. While CVD and cancer have typically been treated as separate disease processes, these results highlight the importance of modifiable risk factors for CVD and cancer prevention. They also demonstrate the clinical utility of the PCE for the synergistic risk stratification of both CVD and cancer, which are the two leading causes of death in developed countries. Further research is needed to understand how the treatment of shared modifiable risk factors via currently available and novel CVD therapies may reduce the morbidity and mortality from both CVD and cancer.

## Declaration of Competing Interest

The authors of this manuscript do not have any relevant conflicts of interest.

## Appendix Supplementary materials

Image, application 1
