# Supplementary material for: Pooled Cohort Equations and the competing risk of cardiovascular disease versus cancer: Multi-Ethnic study of atherosclerosis
Source: Am J Prev Cardiol. 2021 Jun 14;7:100212. doi: 10.1016/j.ajpc.2021.100212 (PMC8387297; doi:10.1016/j.ajpc.2021.100212)
Supplement: Supplementary file 1 [file mmc1.docx]

**Supplementary Material**

Supplemental Table 1 –Incidence rate per 1,000 person-years follow for cardiovascular disease versus cancer stratified by atherosclerotic cardiovascular disease risk groups

|  | **10-Year ASCVD Risk** | | |
| --- | --- | --- | --- |
|  | **<7.5%** | **7.5-<20%** | **≥20%** |
| **Cardiovascular** | 3.3 | 11.5 | 22.8 |
| **Cancer** | 4.8 | 8.6 | 13.6 |

*ASCVD: Atherosclerotic cardiovascular disease

Supplemental Table 2 – Cause-specific hazard for the competing risk of cardiovascular disease versus cancer stratified by atherosclerotic cardiovascular disease group stratified and age

|  | **Age <65 years** | | |  | **Age ≥65 years** | | |
| --- | --- | --- | --- | --- | --- | --- | --- |
|  | **<7.5%** | **7.5-<20%** | **≥20%** |  | **<7.5%** | **7.5-<20%** | **≥20%** |
| **Cardiovascular** | Reference | 4.04 (3.09-5.27) | 6.97 (4.69-10.37) |  | Reference | 1.58 (0.97-2.59) | 3.35 (2.08-5.40) |
| **Cancer** | Reference | 1.67 (1.27-2.19) | 2.17 (1.29-3.67) |  | Reference | 1.91 (1.12-3.26) | 2.94 (1.74-4.98) |

Model 1 –Lipid lowering medication use, body mass index, income, education

Supplemental Figure 1 – Sex-specific proportion of incident cancer in the Multi-Ethnic Study of Atherosclerosis by type.

**Men Women**


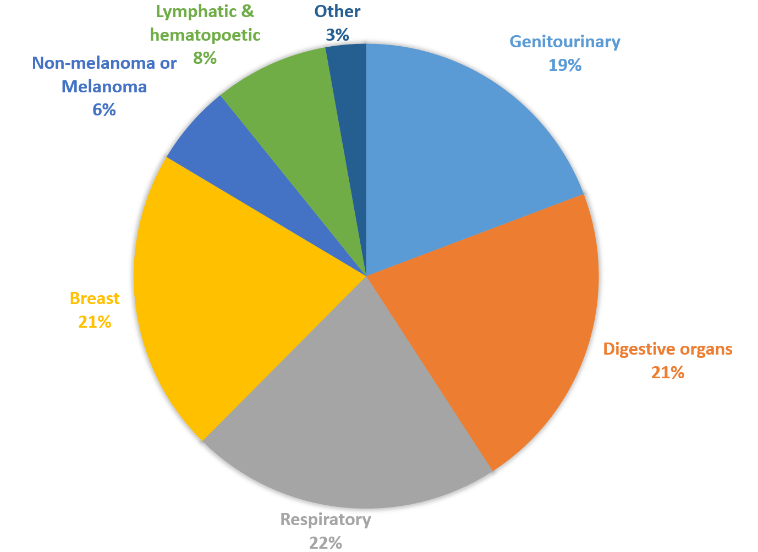

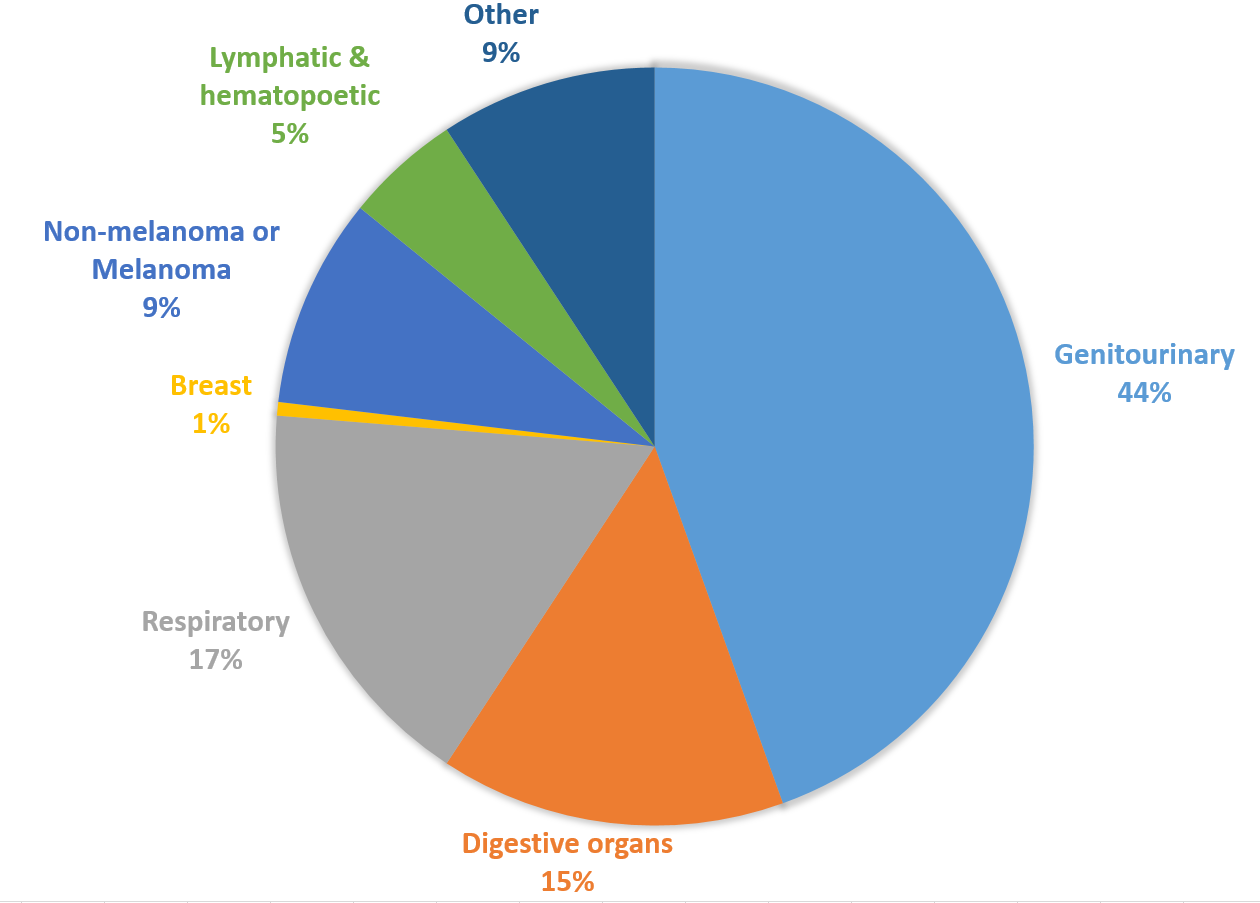


Genitourinary: prostate, uterine, bladder, kidney, ovarian, cervical

Digestive organs: colorectal, liver, pancreas, other gastrointestinal,
